# MicroRNA expression analysis in high fat diet-induced NAFLD-NASH-HCC progression: study on C57BL/6J mice

**DOI:** 10.1186/s12885-015-2007-1

**Published:** 2016-01-05

**Authors:** Alessandra Tessitore, Germana Cicciarelli, Filippo Del Vecchio, Agata Gaggiano, Daniela Verzella, Mariafausta Fischietti, Valentina Mastroiaco, Antonella Vetuschi, Roberta Sferra, Remo Barnabei, Daria Capece, Francesca Zazzeroni, Edoardo Alesse

**Affiliations:** Department of Biotechnological and Applied Clinical Sciences, University of L’Aquila, via Vetoio - Coppito 2, 67100 L’Aquila, Italy; S. Salvatore Hospital, Unit of Laboratory Medicine, L’Aquila, Italy

**Keywords:** microRNA, NAFLD, NASH, HCC, High fat diet, Low fat diet

## Abstract

**Background:**

Hepatocellular carcinoma (HCC) is the most common malignant tumor of the liver. Non-alcoholic fatty liver disease (NAFLD) is a frequent chronic liver disorder in developed countries. NAFLD can progress through the more severe non alcoholic steatohepatitis (NASH), cirrhosis and, lastly, HCC. Genetic and epigenetic alterations of coding genes as well as deregulation of microRNAs (miRNAs) activity play a role in HCC development. In this study, the C57BL/6J mouse model was long term high-fat (HF) or low-fat (LF) diet fed, in order to analyze molecular mechanisms responsible for the hepatic damage progression.

**Methods:**

Mice were HF or LF diet fed for different time points, then plasma and hepatic tissues were collected. Histological and clinical chemistry assays were performed to assess the progression of liver disease. MicroRNAs’ differential expression was evaluated on pooled RNAs from tissues, and some miRNAs showing dysregulation were further analyzed at the individual level.

**Results:**

Cholesterol, low and high density lipoproteins, triglycerides and alanine aminotransferase increase was detected in HF mice. Gross anatomical examination revealed hepatomegaly in HF livers, and histological analysis highlighted different degrees and levels of steatosis, inflammatory infiltrate and fibrosis in HF and LF animals, demonstrating the progression from NAFLD through NASH. Macroscopic nodules, showing typical neoplastic features, were observed in 20 % of HF diet fed mice. Fifteen miRNAs differentially expressed in HF with respect to LF hepatic tissues during the progression of liver damage, and in tumors with respect to HF non tumor liver specimens were identified. Among them, miR-340-5p, miR-484, miR-574-3p, miR-720, whose expression was never described in NAFLD, NASH and HCC tissues, and miR-125a-5p and miR-182, which showed early and significant dysregulation in the sequential hepatic damage process.

**Conclusions:**

In this study, fifteen microRNAs which were modulated in hepatic tissues and in tumors during the transition NAFLD-NASH-HCC are reported. Besides some already described, new and early dysregulated miRNAs were identified. Functional analyses are needed to validate the results here obtained, and to better define the role of these molecules in the progression of the hepatic disease.

**Electronic supplementary material:**

The online version of this article (doi:10.1186/s12885-015-2007-1) contains supplementary material, which is available to authorized users.

## Background

Hepatocellular carcinoma (HCC) is the most frequent liver tumor and the third cause of cancer mortality worldwide [1]. HCC etiopathogenesis is mainly related to viral infections (HBV, HCV) [2], aflatoxin B1 and tobacco exposure [3, 4], or chronic alcohol consumption [5]. Deregulation at the level of several key signal transduction pathways (such as Wnt/β-catenin, MAPK, JAK-STAT, p53) have been extensively described in HCC pathogenesis [6].

Non alcoholic fatty liver disease (NAFLD) is the most frequent liver disorder in western countries and occurs in individuals who do not abuse alcohol. NAFLD can be due to higher fat intake with diet, “de novo” lipogenesisis, or adipose tissue lipolysis increase [7]. It is characterized by accumulation of triglycerides within hepatocytes (steatosis), attributable to an imbalance between storage and removal of lipids, and it is associated with obesity and metabolic syndrome [8]. In a number of cases, NAFLD progresses from simple steatosis to non alcoholic steatohepatitis (NASH), a form of hepatic damage characterized by the recruitment of pro-inflammatory immune cells, and lastly toward cirrhosis and hepatocellular carcinoma [7]. It has been calculated that a percentage variable between 4 and 22 % of HCC cases can be ascribed to NAFLD [8]. However, molecular mechanisms responsible for NAFLD-NASH-HCC progression are not fully understood.

MicroRNAs (miRs, miRNAs) are short non-coding molecules able to regulate gene expression at the post-transcriptional level. MicroRNAs are involved in fundamental cellular processes, such as growth, proliferation and differentiation, apoptosis, metabolism, oncogenesis and metastasis [9, 10]. Many miRNAs have been described in the initiation and progression of liver cancer [11, 12]. Several down-regulated (i.e. miR-1, miR-7, miR-34a, miR-122, miR-125b, miR-200) or up-regulated (i.e. miR-17, miR-18, miR-19, miR-155, miR-93, miR-221/222) miRNAs have been identified as tumor suppressor or oncomirs, respectively, by targeting and regulating genes involved in cell proliferation, apoptosis, angiogenesis and metastasis [13]. Several studies have furthermore shown expression level dysregulation and modulation of microRNAs in NAFLD, NASH, and then HCC. Among them, miR-122, miR-21, miR-155, miR-23a, miR-143, whose target genes have been characterized in both NAFLD (i.e. *PPARα, PTEN C/EBPβ, ORP8, G6PC*) and HCC (i.e. *CCNG1, IGF-1R, ADAM17, PTEN, SOCS1, C/EBPβ, FNDC3B*) [14]. In addition, miRNAs have been described to be modulated even in steatosis/NASH (i.e. miR-155, miR-370, miR-34a, miR-200a/b, miR-99a/b), fibrosis (i.e. miR-200a/b, miR-221/222, miR-34a, miR-16, miR-99b), cirrhosis (i.e. miR-34a, miR-21, miR-31, miR-181b), and HCC (i.e. miR-16, miR-33, miR-21, miR-31, miR-181a/b, miR-99a, miR-200a/b) [15]. However, miRNAs specifically involved in the progression of liver disease are not fully characterized. Therefore, to better define and identify microRNAs playing a pivotal role in this process, we analyzed in a time-dependent and dynamic manner the expression levels of miRNAs in livers from a long term high fat diet fed C57BL/6J mouse model, with the purpose to put into relation the expression levels of miRNAs with the progression of the liver’s injury.

## Methods

### Mouse strain and housing

C57BL/6J mice were purchased from Charles Rivers Laboratories (France) and maintained at 21 °C on a 12 h light–dark cycle. Twenty days old male mice obtained from the established colony were randomly split in 3 groups (10 animals each), and fed with a high fat diet (5.56 Kcal/g, fat 58 Kcal%, whose coconut oil hydrogenated 54 %; carbohydrate 25.5 Kcal%) (D12331, OpenSource, Research Diets) for 3, 6, and 12 months. Analogously, 3 groups of control animals (10 animals each) were fed with the control low fat diet (4.07 Kcal/g, fat 10.5 Kcal%; carbohydrate 73.1 Kcal%, whose sucrose 60 %) (D12329, Open Source, Research Diets). Mice were weighed at approximately one-month intervals and periodically analyzed for signs of disease or morbidity. Mice were sacrificed by CO_2_ asphyxiation, weighed, and head-to-tail measured. Laparotomy was then performed, and the liver was visualized and rapidly excised, weighed and photographed. The following parameters were considered: liver appearance, color and weight. Liver tumors were counted and measured. All experimental procedures involving animals and their care were performed in conformity with national and international laws and policies (European Economic Community Council Directive 86/609, OJ 358, 1 Dec 12, 1987; Italian Legislative Decree 116/92, Gazzetta Ufficiale della Repubblica Italiana n. 40, Feb 18, 1992; National Institutes of Health Guide for the Care and Use of Laboratory Animals, NIH publication no. 85–23, 1985). The project was approved by the Italian Ministry of Health and the internal Committee of the University of L’Aquila. All efforts were made to minimize suffering.

### Assessment of microscopic hepatic lesions

Specimens obtained from livers were washed in PBS and immediately immersed in 10 % formalin in phosphate buffered saline (PBS) (pH 7.4), then standard procedures for paraffin embedding were performed. Serial 3 μm sections were stained with Hematoxylin and Eosin (H&E) to assess the liver general architecture and inflammation. Masson’s trichrome stain was also performed in order to detect connective tissue and fibrosis. The stained sections were then observed by using Olympus BX51 Light Microscope (Olympus, Optical Co., Ltd, Tokyo, Japan).

### Biochemical assays

After sacrifice, blood was collected in heparin by cardiac puncture, and plasma was immediately recovered and stored at −80 °C for subsequent analyses. A panel of biomarkers for characterizing the metabolic features of liver disease was analyzed by using Architect system and kits (Abbott Diagnostics), according to the manufacturer’s instructions.

### RNA extraction

Liver tissues and excised tumors were sectioned and stored in RNAlater® stabilization solution (Ambion) at −80 °C. RNA was extracted from whole hepatic specimens and tumors by using miRVana™ microRNA isolation kit (Life Technologies), according to the manufacturer’s instructions.

### Real-time quantitative PCR

Identical amounts of total RNAs extracted from animals belonging to the same experimental group were pooled together and subjected (700 ng per RNAs’ pool) to RT-PCR by using the TaqMan MicroRNA reverse transcription kit and the Megaplex RT primer pool (Life Technologies). Subsequently, microfluidic Rodent MicroRNA arrays v3.0 (Life Technologies) were used, according to the manufacturer’s instructions. Three replicates for each pooled sample were analyzed. MicroRNAs’ expression levels were evaluated by comparative assay. Samples were analyzed on a ViiA7 instrument (Life Technologies) and data were processed by ViiA7 software (Life Technologies). ΔΔCt method was used to determine the relative miRNAs’ expression levels. Mamm U6 was used as endogenous control. Global normalization analysis was also performed (Expression Suite, Life Technologies). Some specific MicroRNA Assays (Life Technologies) were performed on each single sample (3 replicates) in order to assess the miRNAs’ expression at the individual level. Further data analysis was carried out by using Expression Suite (Life Technologies) or GraphPad Prism (GraphPad software).

## Results

### Diet-induced obesity

**Fig. 1:**
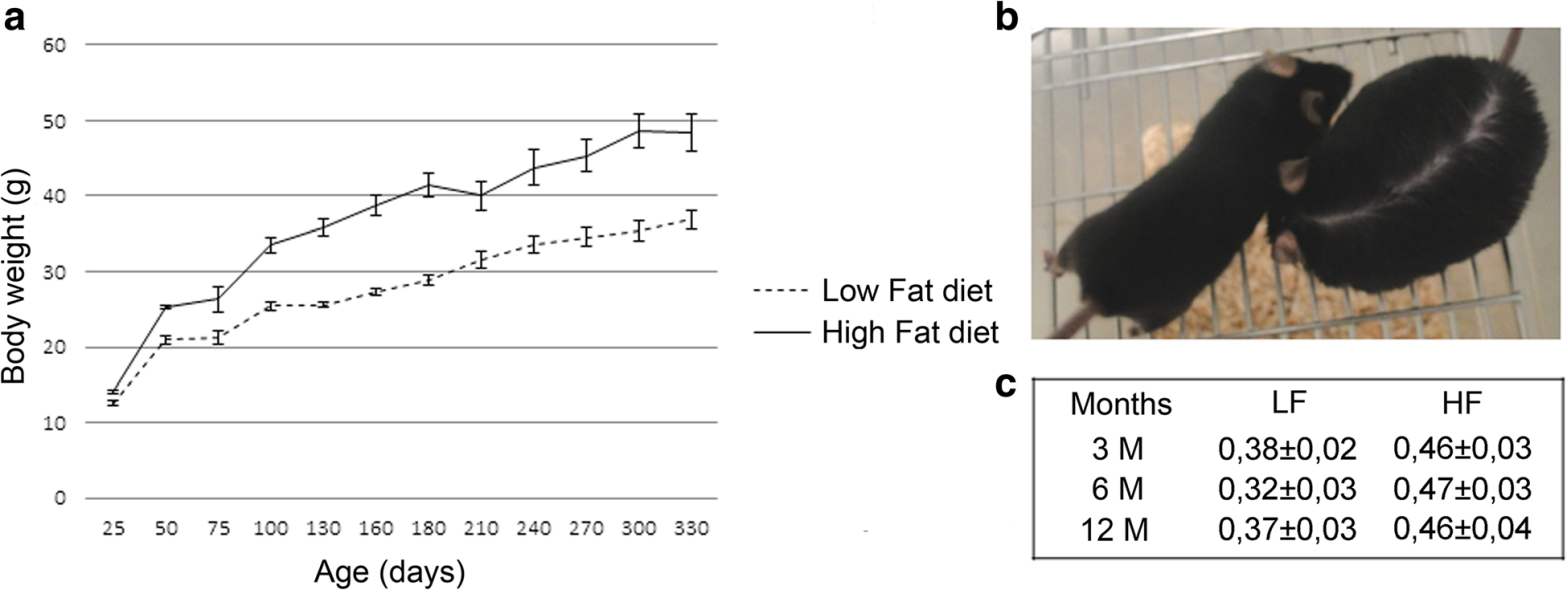
Body weight patterns. **a** Mice were high fat (HF) or low fat (LF) diet fed, and weighed at the indicated time points. Values are means of 10 mice ± SEM. **b** Representative picture of a 6 months LF (*left*) and HF (*right*) diet fed mouse. **c** Mean of body mass index values ± SEM

C57BL/6J male mice and, with lower evidence females, have been already described to be predisposed and susceptible to NAFLD and diet-induced obesity with respect to other strains (A/J), in both short and long-term fatty diet fed models [16, 17]. In our model, we analyzed the effects of a HF diet on liver disease induction. For this purpose, C57BL/6J mice groups were treated for different times with HF (majority of calorie count due to hydrogenated coconut oil) or LF (majority of calorie count due to sucrose) high-calorie diets. Body weights’ patterns of HF and LF diet-treated animals are reported in Fig. 1a. HF mice developed significant weight increase, as detected after 3, 6 and 12 months (*P*_3, 6, 12M_ < 0.001), and associated obesity (Fig. 1b), further confirmed by BMI values (Fig. 1c). In particular, an overt accumulation of subcutaneous, visceral and thoracic fat was detected in HF mice (data not shown).

### Histological liver features

**Fig. 2:**
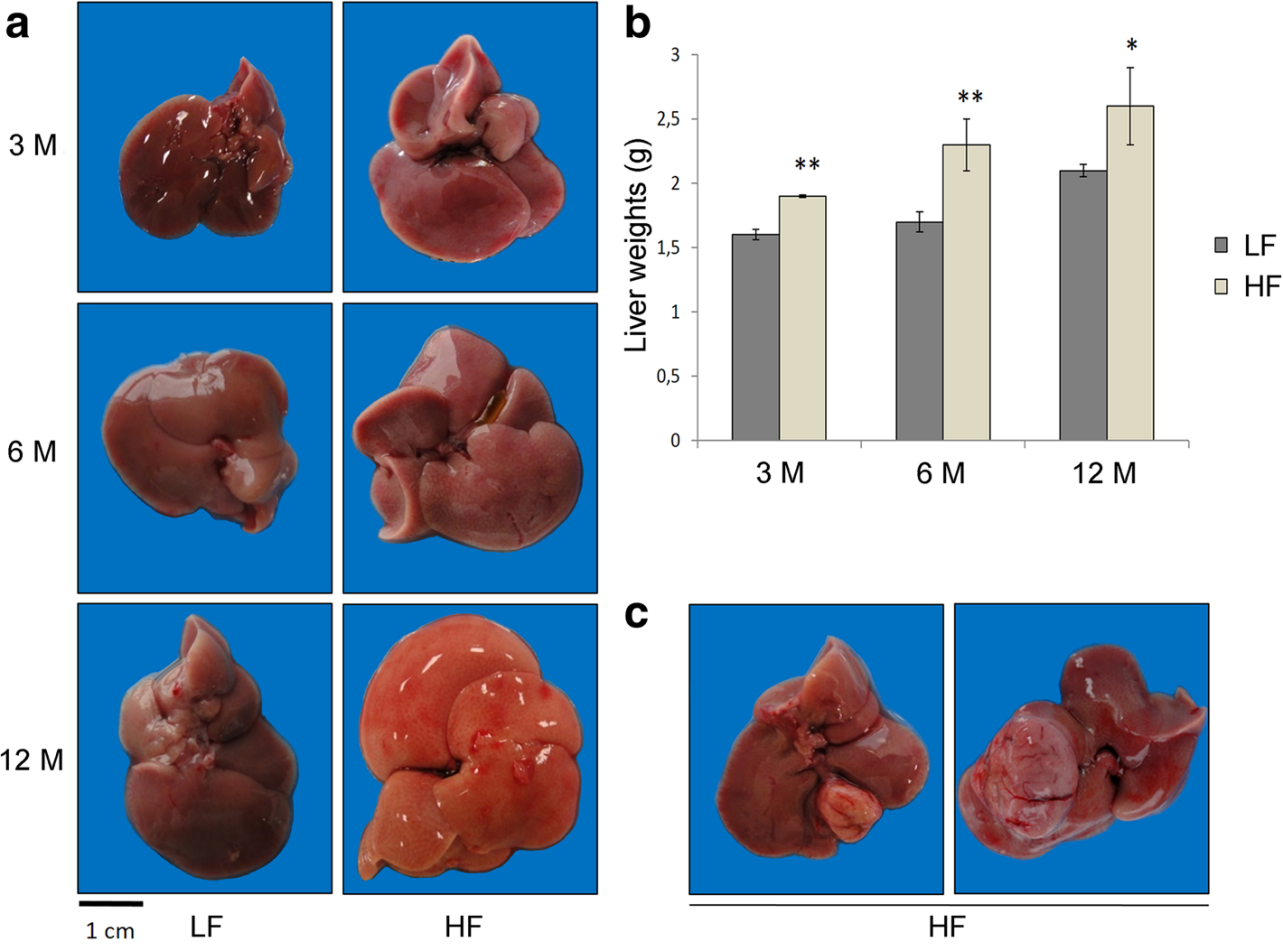
Livers from HF and LF diet fed mice. **a** Livers from 3, 6, 12 months LF (*left*) and HF (*right*) diet fed mice. **b** Liver weights, expressed as mean ± SEM. Statistical significance is indicated as follows: **, *P* < 0.08; *, *P* = 0.05. **c** Macroscopic nodules in 12 months HF diet fed mice

**Fig. 3:**
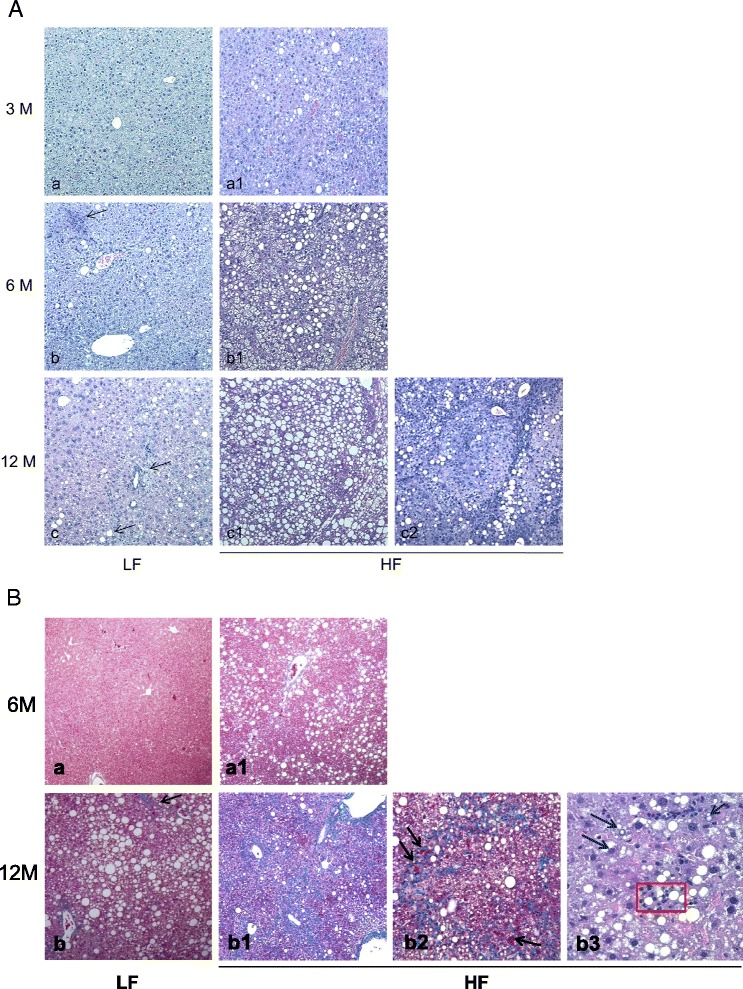
Histopathological features of hepatic tissues. **A** Histopathological features of hepatic tissues from 3 (*a, a1*), 6 (*b, b1*), 12 (*c, c1, c2*) months LF (*left*) and HF (*right*) mice (H&E staining; original magnification 10X). The microphotographs, from LF mice, show a normal liver architecture (*a*), scattered inflammation (*b, arrow*) and simple steatosis with mild inflammation (*c, arrows*). A wide spectrum of liver damage ranging from simple steatosis (*a1*) to mild steatosis (*b1*) and a severe steatosis with massive inflammation (*c1, c2*) are shown in microphotographs from HF mice. **B** Fibrosis is not evident in 6 months LF (*a*) and HF (*a1*) mice (Masson’s trichrome staining, original magnification, 10X). Mild fibrosis appears after 12 months in LF mice (b, arrow, original magnification, 10X), whereas 12 months HF mice show more severe fibrosis (b1, original magnification 10X), often organized in irregular thin trabeculae that border nodules with a variable number of small microscopic arteries (*arrows*), and a disarrangement of normal hepatic architecture with an increase of cell density and frequent steatosis (*b2*). Moreover, there is a certain degree of cellular atypia, rare pseudoglandular structures and steatosis (b3, H&E original magnification 40X, red box and arrows respectively). These aspects are common features of dysplastic nodules or early HCC

**Fig. 4:**
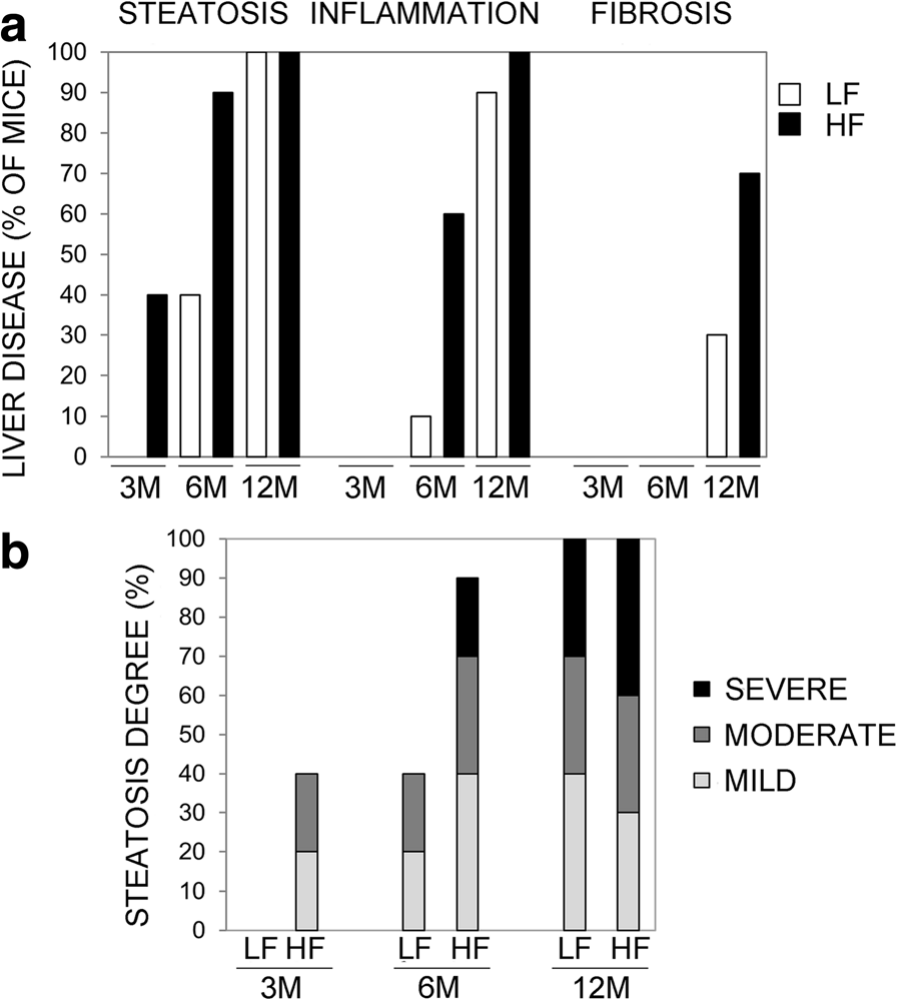
Progression of liver disease. **a** Percentage of HF/LF mice showing steatosis, hepatic inflammation and fibrosis. **b** Degree of steatosis in HF and LF diet fed animals

Gross anatomical examination revealed, in livers from HF animals, hepatomegaly as well as paler color (Fig. 2a). Significant weight increase of HF livers was also detected (Fig. 2b). Two voluminous macroscopic nodules (1.5x1.3x1 and 0.7x0.6x0.5 cm in dimensions) (Fig. 2c) were observed in 2 HF mice (20 %) after 12 months of fatty diet regimen. No nodular formations were detected in the LF groups. Histomorphological analysis showed a wide spectrum of liver damage ranging from simple steatosis, consisting of isolated fat deposition in hepatocytes from 3 months HF mice (Fig. 3a, a1), more pronounced steatosis in 6 months animals (Fig. 3a, b1), and steatohepatitis in 12 months HF mice (Fig. 3a, c1, c2). Inflammatory infiltrate was characterized by lymphocytes, plasma cells, macrophages and polymorphonuclear leucocytes (PMN) (Additional file 1: Figure S1). Twelve months HF livers were also characterized by fibrosis (Fig. 3b, b1), and disarrangement of normal hepatic architecture with increase of cell density and frequent steatosis (b2). Moreover, a certain degree of cellular atypia, rare pseudoglandular structures and steatosis can be detected (b3, H&E original magnification 40X, arrows and red box, respectively). The latter aspects are common features of dysplastic nodules or early HCC. The described traits demonstrate the progression of liver damage through NAFLD, NASH, fibrosis and HCC. On the other hand, LF diet fed mice showed normal liver architecture after 3 months (Fig. 3a, a), scattered hepatic inflammatory cells in a small percentage of animals after 6 months (Fig. 3a, b, arrow) and accumulation of triglycerides in combination with hepatic inflammation after 12 months of LF diet treatment (Fig. 3a, c, arrows). Less severe fibrosis was detected in LF mice after 12 months (Fig. 3b, b). No fibrosis was detected in HF and LF mice after 3 and 6 months (Fig. 3b, a, a1) of treatment. In summary, concerning the progression of liver disease, steatosis, with ascending degree of severity, was found in 40 %, 90 % and 100 % of 3, 6 and 12 months HF diet fed mice (Fig. 4a, b). Inflammation was evident in 60 % of 6 months and 100 % of 12 months HF mice, whereas fibrosis was detected in 70 % of animals just after 12 months (Fig. 4a). Contextually, in LF mice, steatosis was not evidenced after 3 months, but was detected in 40 % and 100 % of animals after 6 and 12 months (Fig. 4a), albeit with lower degree of severity with respect to the corresponding HF groups (Fig. 4b). Inflammation, at the same way, was undetectable after 3 months and revealed in 10 % and 90 % of mice after 6 and 12 months of LF diet administration (Fig. 4a). Fibrosis was detected in 30 % of LF animals after 12 months (Fig. 4a). Significant cirrhosis was not evidenced by any mouse belonging to both HF and LF groups.

### Clinical chemistry assays

In order to assess the evolution of the hepatic damage and the relative metabolic features, a panel of plasma biomarkers was examined in non-fasting mice through the experimental time points (Table 1). Significant increase of cholesterol (CHOL), as well as high density lipoproteins (UHDL), low density lipoproteins (DLDL), and triglycerides (TRIG) was detected in HF mice after 3, 6, 12 months (UHDL) or 3, 6 months of treatment (CHOL, DLDL, TRIG). Alanine aminotranferase (ALT) was significantly increased after 3 and 12 months of HF diet administration. ALT increase was also revealed in HF mice after 6 months, but no statistically significant difference was evidenced. Data obtained indicate metabolic dysfunctions, development and progression of liver injury, confirming the role of HF metabolic regimen. Similar results were obtained in studies on short term lard-containing HF diets fed mice, where LDL, HDL, AST, ALT, TRIG significant increase was detected [18–20]. Significant ALT increase was also described by Hill-Baskin et al. [17]. Levels of ALT, AST, and AST/ALT ratio have been taken into consideration as possible markers for NAFLD and its progression, although liver biopsy remains the gold standard for diagnosis [21, 22].

**Table 1:** Plasma biomarkers in HF and LF diet fed animals. Values are mean ± SEM. *P* < 0.05 was considered for statistically significant differences (marked with an asterisk)

Marker	3M HF	3M LF	P_3M_	6M HF	6M LF	P_6M_	12M HF	12M LF	P_12M_
ALT (U/l)	43.2 ± 3.5	23.3 ± 3	0.001*	70.5 ± 11.7	50.2 ± 18.9	0.16	89 ± 23	45.6 ± 6.7	0.03*
AST (U/l)	204.8 ± 59.3	155.3 ± 46.2	0.27	135.33 ± 20.4	200.78 ± 50.7	0.33	156.8 ± 24.2	202.9 ± 32.1	0.12
GLUC (mg/dl)	491.6 ± 56.3	425 ± 30.2	0.19	452.33 ± 13.8	359.56 ± 25.4	0.07	388.5 ± 32.9	398.2 ± 30.3	0.39
TRIG (mg/dl)	139.6 ± 8.7	87.2 ± 6.8	<0.001*	122.6 ± 10.4	72.4 ± 5.2	0.002*	100.7 ± 7.6	82.2 ± 6.2	0.06
CHOL (mg/dl)	214.1 ± 10.6	134 ± 7.9	<0.001*	233.78 ± 10.1	119.33 ± 8.8	<0.001*	208.3 ± 18.6	173.2 ± 10.1	0.06
DLDL (mg/dl)	10.1 ± 0.87	7.2 ± 0.6	0.008*	14.3 ± 1.6	8 ± 1.1	0.005*	15.1 ± 1.7	12.6 ± 1	0,07
UHDL (mg/dl)	112.6 ± 4	73.2 ± 3.8	0.002*	107.33 ± 3.3	61 ± 3.8	<0.001*	100.1 ± 6.5	80 ± 2.4	0.01*

*ALT* alanine aminotransferase, *AST* aspartate aminotransferase, *GLUC* glucose, *TRIG* triglycerides, *CHOL* cholesterol, *DLDL* direct low density lipoprotein, *UHDL* ultra high density lipoprotein assay

### MicroRNA analysis

**Fig. 5:**
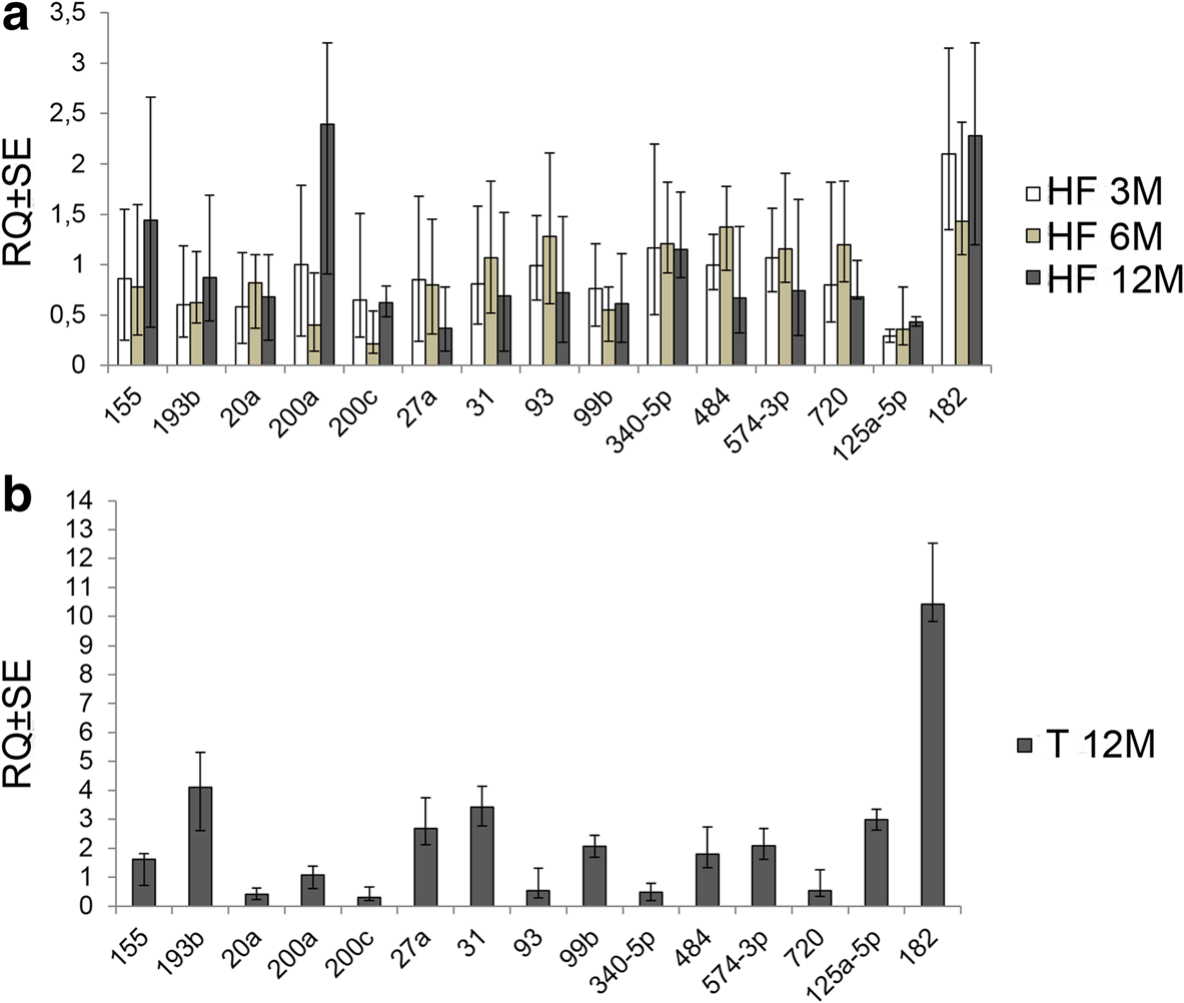
MiRNAs differentially modulated during the progression of the hepatic damage. **a** RQ (relative quantification) values ± SE (Y axis) obtained by comparing HF to LF pooled RNAs from hepatic tissues. **b** RQ values ± SE (Y axis) of pooled RNAs from tumor tissues with respect to pooled RNAs from HF hepatic non-tumor tissues. Results are from 3 replicates. MammU6 was used as endogenous control

**Fig. 6:**
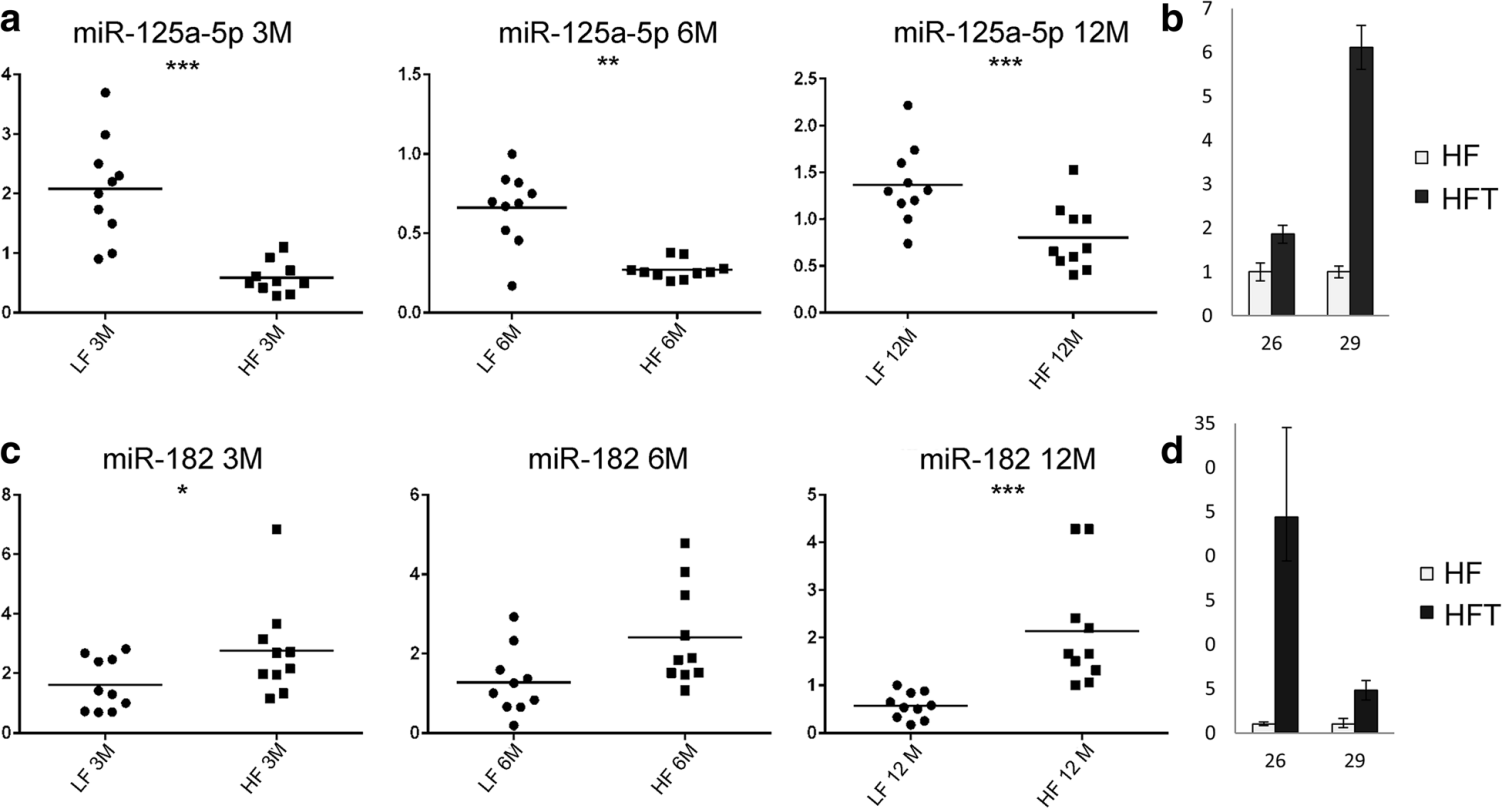
Differential expression of miR-125a-5p and miR-182 in livers and tumors, at the individual level. (**a**) (**c**) RQ (relative quantification) values ± SE (Y axis) in LF and HF mice (X axis) after diet treatment for 3, 6, and 12 months. MammU6 was used as endogenous control. RQ values were calculated with respect to one reference LF mouse in each experimental group (ID#: 2LF 3 M; 11LF, 6 M; 21LF, 12 M). Statistical significance is marked as follows: *, *P* < 0.05; **, *P* < 0.004; ***, *P* < 0.002 (**b**) (**d**) RQ values ± SE (Y axis) obtained by comparing tumors (HFT, black) vs. paired non tumor HF livers (*light gray*). 26 and 29 are ID numbers of mice with tumors

A panel of miRNAs was subjected to analysis during the progression of the liver disease. Among them, some miRNAs revealed a modulation during the transition of the hepatic damage. Results are shown in Fig. 5. MiRs’ differential expression was evaluated by comparing pooled mRNAs from 3, 6, 12 months HF vs LF liver tissues (Fig. 5a) and pooled mRNAs from tumors vs pooled mRNAs from 12 months HF non-tumor tissues (Fig. 5b). MammU6 was used as endogenous control. Some miRNAs were overexpressed in tumors (miR-155, miR-193b, miR-27a, miR-31, miR-99b, miR-484, miR-574-3p, miR-125a-5p, miR-182), whereas others displayed down-regulation (miR-20a, miR-200c, miR-93, miR-340-5p, miR-720) or a comparable level of expression (miR-200a) with respect to non tumor tissues. Depending on the treatment’s duration, different modulation of miRs’ expression was detected in HF tissues during the progression of the hepatic damage (Fig. 5a). Mir-155 level increased after 12 months of HF treatment; miR-193b, which was down-regulated after 3 months of treatment, showed weak ascending expression, whereas miR-31 and miR-93 revealed fluctuant levels during the treatment, with slight down-regulation after 12 months. MiR-20a, miR-200c, miR-27a, miR-99b displayed a global, more or less marked, down-regulation during the treatment. MiR-200a revealed a modulation, being down-regulated after 6 months and over-expressed after 12 months of HF diet. MiR-340-5p, miR-484, miR-574-3p, and miR-720 showed fluctuant levels of slight down-regulation or over-expression during the treatment. MiR-182 showed marked over-expression, as detected already after 3 months of treatment, whereas miR-125a-5p was always down-regulated in HF compared to LF tissues. Similar results were also obtained by analyzing data using global normalization (Additional file 2: Figure S2). To assess the strength of data shown in Fig. 5, the expression levels of miR-125a-5p and miR-182 were analyzed in individual livers from HF and LF diet fed mice through experimental time points and in tumors. MiR-125a-5p and miR-182 expression was evaluated by taking into consideration a LF reference sample belonging to the same group (Fig. 6). Significant down-regulation of miR-125a-5p was detected in HF mice after 3 months of HF diet regimen and confirmed after 6 months (Fig. 6a). Twelve months HF diet-treated mice showed, at the same way, significant down-regulation of miR-125a-5p (Fig. 6a). Conversely, miR-125a-5p over-expression was detected in tumors with respect to paired HF non tumor tissues (Fig. 6b). Significant miR-182 over-expression was detected in 3 months and, although less pronounced and not statistically significant, in 6 months HF diet fed mice (Fig. 6c). Significant miR-182 over-expression was observed in 12 months HF mice (Fig. 6c). Over-expression was further confirmed in tumors from 12 months treated animals (Fig. 6d).

## Discussion

Nonalcoholic fatty liver disease is the most frequent chronic liver disease in western countries. It exhibits intra-hepatic fat accumulation and can progress through the more severe nonalcoholic steatohepatitis, leading, in a percentage of cases, to end-stage cirrhosis and HCC. Currently, some serum biomarkers are taken into consideration to diagnose and predict the progression of the disease, despite their limited prognostic usefulness, sensitivity, and tissue specificity. Several biomarkers, such as alpha-fetoprotein, alone or also in combination with osteopontin, glypican-3, laminin, VEGF (vascular endothelial growth factor), or hyaluronic acid, have been used to assess, without particularly significant results, HCC occurrence in NAFLD patients [23–27]. Liver biopsy is still the most accurate procedure to diagnose and provide information about staging of liver disease, although studies have demonstrated that patients with initial NAFLD clinical manifestation and diagnosis do not develop HCC and that a regression may be also possible in pre-cirrhotic stages of the disease [28]. Therefore, there is an urgent need to identify new diagnostic and prognostic markers able to follow the progression through NAFLD-NASH and HCC initiation and development.

MicroRNAs are short endogenous molecules which act in post-transcriptional gene regulation. Due to their role and structure, scientific evidences highlight the promising value of microRNAs as biomarkers at the diagnostic and prognostic level. In this study, we used a mouse model predisposed to NAFLD and obesity to analyze the progression of high-fat diet induced liver disease through NAFLD-NASH up to HCC initiation and development. Depending on the treatment’s duration, HF-fed animals showed an increase of body and liver weights, degree of steatosis, presence of inflammatory infiltrate and fibrosis, demonstrating the progression of liver disease. As described, the LF group showed pathological features similar to the HF, which, however, appeared later and with lower severity. This could be explained by the fact that the control LF diet here used, with higher caloric content than a standard diet, is formulated low in fat, but high in sucrose. Previous studies have discussed the role of high-carbohydrate diets on lipid accumulation and the effects of chronic fructose consumption on different tissues: in liver, inflammation, dyslipidemia, and steatosis have been described [29–31]. This could trigger the *de novo* lipogenesis process, with delayed lipid accumulation and cellular damage in livers in comparison to that observed in HF mice. A recent work, performed on 15 weeks-old high fructose or sucrose diet fed C57BL/6 mice, showed fatty infiltration of necroinflammatory areas, which are characteristic features of the transition to NASH, enhanced lipogenesis, gluconeogenesis and anti-oxidant imbalance, demonstrating an adverse effect of fructose or sucrose-rich diets on liver [32]. Biochemical assays highlighted increasing values of plasma biomarkers in HF animals, characterizing the presence of metabolic dysfunctions and liver damage. No particular evidence of cirrhosis was detected, and a percentage of HF fed mice (2/10) developed tumors after 12 months. Fifteen miRs resulted differentially expressed in livers, by comparing HF- and LF-diet treated animals, and in tumors with respect to non tumor HF liver tissues, providing evidence of their modulation during the progression of diet-induced liver damage. As summarized in Table 2, some among them were already described in NAFLD, NASH, fibrosis or HCC, whereas others are for the first time here identified. MiR-155, whose expression increased after 12 months HF diet treatment, resulted over-expressed in tumors and in HF tissues with respect to LF. Previous studies have demonstrated that miR-155 plays an important role in hepatic lipid metabolism, has a protective role against HF diet-induced non alcoholic liver steatosis [33], and was found to be up-regulated in NASH models of methyl-deficient diet, in HCC induced by choline-deficient and amino acid-defined diet, and in primary human HCC [34, 35]. Moreover, it has been demonstrated that miR-155 deficiency can attenuate steatosis and fibrosis [36]. In addition, anti-miR-155 has shown in vitro and in vivo potential therapeutic efficacy, by restoring the expression of *C/EBPβ* and *FOXP3* [37].

**Table 2:** Dysregulated miRNAs and their involvement in liver disease. ICC intrahepatic cholangiocarcinoma, HNSCC head and neck squamous cell carcinoma

MicroRNA	Liver disease	Target gene(s)
miR-155	Protective role against non alcoholic steatosis [33]	LXR-α[33] C/EBPβ, FOXP3 [35–37]
	Up-regulated in NASH [34]	
	Up-regulated in HCC [35]	
	MiR-155 deficiency attenuates steatosis and fibrosis [36]	
miR-193b	Down-regulated in HBV+ HCC [43]	CCND1, ETS1 [43]
		NF1 (HNSCC) [46]
		Smad3 (glioma) [47]
miR-20a	Down-regulated in HCC [49]	Mcl-1 [49]
miR-200a	Up-regulated in NAFLD [51]	ZEB1, ZEB2 [54]
	Down-regulated in HCC [52]	
miR-200c	Up-regulated in NAFLD [51]	ZEB1, ZEB2, NCAM1 [54]
	Down-regulated in HCC and ICC [54]	
miR-27a	Up-regulated in HBV+ HCC [55]	RXRα, PPARα/γ, FASN, SREBP1, SREBP2 [58, 59]
miR-31	Up-regulated in fibrosis [60]	FIH1 [60]
	Up-regulated in HCC [17, 53]	
miR-93	Up-regulated in HCC [61, 62]	PTEN, CDKN1A [62]
miR-99b	Up-regulated in HCC [69]	mTOR (pancreatic cancer) [66]
		CLDN11 [69]
miR-125a-5p	Down-regulated in HCC [89]	SIRT7 [89]
	Biomarker in liver disease [93]	
miR-182	Up-regulated in NAFLD-fibrosis [98]	FOXO3 [98]
		MTSS1 [94], Cebpa [95],
	Up-regulated in HCC [94–97]	EphrinA5 [96], FOXO1 [97]

MiR-193b was down-regulated after 3 and 6 months of HF regimen and revealed over-expression in tumor samples. The role of this miR in carcinogenesis is quite controversial: miR-193b was described as a tumor suppressor and appeared down-regulated in several cancers, such as melanoma, breast, prostate carcinoma, and human HCC tissues, mainly HBV-positive [38–43]. In vitro and in vivo experimental data demonstrated that miR-193b directly targeted *CCND1* (*cyclin D1*) and the transcription factor *ETS1* [43]. In a study on two HF diet fed mouse models, showing marked susceptibility (C57BL/6J) or resistance (Balb/c) to NAFLD and insulin resistance phenotype, significant up- or down-regulation of key genes which may be involved in homeostatic adaptation to HF regimen has been detected. Among them, are *CCND1* and *ETS1*, whose up-regulation was detected in both strains or in C57BL/6J alone, respectively [44]. This evidence could be in agreement with miR-193b down-regulation detected in our model during the first 6 months of HF diet treatment. With this regard, *ETS1*/miR-193b 3′UTR alignment can be identified in *Mus musculus* (microrna.org: SVR score −0.121, PhastCons 0.66). On the other hand, miR-193b over-expression was described by Braconi et al. [45] in HCV-positive HCC tissues and cells. MiR-193b over-expression was also detected in head and neck squamous cell carcinoma [46], where *neurofibromin1* (*NF1*) was described as a target, and in glioma [47], where this miR acted as an oncomiR by targeting *Smad3*, one of the major *TGF-β* signaling transducers. Beside, a study on a mouse model demonstrated that forced expression of *Smad3* may reduce liver susceptibility to chemically-induced carcinogenesis by promoting apoptosis through *Bcl-2* transcriptional repression [48]. With this regard, two miR-193b target sites are predicted on mouse *Smad3* 3′-UTR (microRNA.org: miR-SVR score −0.1684 and −0.0002; PhastCons 0.5285 and 0.5702, respectively), leaving hypothesize that miR-193b over-expression could be involved in hepatocarcinogenesis through *Smad3* down-regulation.

MiR-20a was down-regulated in liver tissues and in tumors from HF mice. MiR-20a down-regulation was described in human HCC, where *Mcl-1* (*myeloid cell leukemia sequence 1*), an anti-apoptotic member of *Bcl-2* family, was identified as miR-20a target [49]. In accordance, a miR-20a predicted target site is located on *Mus musculus Mcl-1* 3′UTR (microRNA.org: mirSVR score −0.9773, PhastCons 0.710).

MiR-200a and c, members of the miR-200 family, showed different behavior: after expression level’s decrease (6 months), miR-200a increased during the progression of hepatic damage (12 months), whereas miR-200c revealed a trend of down-regulation during HF diet treatment and in tumors. It is known that miR-200 family plays a role as tumor suppressor by inhibiting epithelial-to-mesenchymal transition (EMT) and repressing cancer stem cells; in addition, its deregulation has been described in several tumor types, including hepatocarcinoma [50]. MiR-200a was found to be up-regulated in NAFLD [51], significantly down-regulated in human HCC samples and, along with miR-200 family members, has been described as a marker able to distinguish between cirrhotic and cancer tissues [52, 53]. MiR-200c was also found to be up-regulated in NAFLD and down-regulated in human HCC as well as in intrahepatic cholangiocarcinoma (ICC) samples [51, 54]. With regard to ICC, Oishi et al. [54] found that miR-200c and miR-141, were negatively correlated with genes involved in the *TGF-β*, *NF-κB* and *Smad* signaling pathway. In addition, these two miRs were able to induce epithelial differentiation and to suppress EMT by inhibition of *ZEB1* and *ZEB2*. The same authors also described *NCAM1*, a known hepatic stem cells marker strictly connected to EMT process, as a miR-200c direct target. Analogously, several miR-200c binding sites are predicted on *Mus musculus ZEB1*, *ZEB2* and *NCAM1* 3′UTR, indicating its putative role in the mouse model here presented.

MiR-27a showed expression decrease, starting faintly after 3 months up to 12 months of HF diet administration. Conversely, it was over-expressed in tumors. Literature data reveal that miR-27a may have an oncogenic role, being up-regulated in HBV-related HCC tissues and HCC cell lines [55], and promoting proliferation in liver cancer cells by diminishing *TGF-β* tumor suppressive activity [56]. MiR-27a was also found in a hypomethylated status which led to its over-expression in HCC cells [57]. MIR-27a was described to be involved in lipid metabolism, by regulating *RXRα*, *PPARα/γ*, *FASN*, *SREBP1*, *SREBP2*, and was able to inhibit HCV replication in human hepatoma cells [58]. Ji et al. [59] demonstrated that miR-27a/b were over-expressed in primary culture activated rat hepatic stellate cells (HSCs). Normal HSCs are in the space of Disse, storing bunches of vitamin A-riching lipid droplets. On the contrary, activated HSCs lose cytoplasmic lipid droplets and trans-differentiate to proliferative, fibrogenic myofibroblasts which play an essential role in liver fibrosis initiation. In the above-mentioned study, miR-27a/b downregulation was demonstrated to be able to activate HSCs to switch to a more quiescent phenotype, with decreased cell proliferation and restored cytoplasmic lipid droplets. Seen in this context, it could be supposed that miR-27a hypoexpression (6M, weak, and 12M) in HF diet model might act as a protective mechanism in limiting the progression of liver damage during the phases of the disease, and, on the other hand, its over-expression in tumors could be associated to promotion of heavier liver injury with consequent HCC initiation.

MiR-31 was detected up-regulated in tumors with respect to livers from 12 months HF mice. MiR-31 up-regulation was also described in human HCC samples and in a similar C57BL/6J high-fat diet fed model [17, 51]. MiR-31 up-regulation was also described in fibrosis [60].

MiR-93 showed slight hypo-expression after 12 months HF diet and resulted down-regulated in HCC. Although, previous reports described an increase of miR-93 level during hepatic tumorigenesis [61], and over-expression in human HCC cell lines and tissues [62], miR-93 down-regulation significantly correlated with worse prognosis in colorectal cancer, where it was described to suppress oncogenesis by regulating *Wnt/β-catenin* pathway [63, 64].

MiR-99b was weakly down-regulated during HF diet administration and, conversely, up-regulated in tumors. MiR-99b was described to contribute to irradiation resistance in human pancreatic cancer by targeting *mTOR* [65], whose activity is also known to play a role in NAFLD-NASH [66–68]. In this context, miR-99b hypoexpression in our model might contribute to induce *mTOR* expression and function in the progression through NAFLD and NASH. A mir-99b/*mTOR* site alignment is also predicted on mouse (mirSVR score, −1.2245; PhastCons score, 0.7484). In a very recent work [69] miR-99b was up-regulated in HCC, where it promoted metastasis by inhibiting *claudin 1* (*CLDN1*). *In silico* analysis displays two predicted miR-99b sites also on mouse *CLDN1* (PhastCons 0.55 and 0.60).

No data are reported about the expression and role of miR-340-5p, miR-484, miR-574-3p, and miR-720 in NAFLD, NASH and HCC tissues. The above-mentioned miRs appear to be up- (miR-484 and miR-574-3p) or down-regulated (miR-340-5p, miR-720) in tumor tissues. Just one study showed miR-574-3p increase in sera from HCC and liver cirrhosis patients [70]. Controversial evidences about the role of those miRNAs in oncogenesis are reported in several studies. MiR-340 has been described as a tumor suppressor in breast [71], NSCLC [72], and melanoma [73]. Significant miR-484 level increase was described in sera from early breast cancer [74] and in melanoma [75], whereas miR-484 down-regulation was displayed in urine from prostate cancer patients [76]. MiR-574-3p was identified over-expressed in plasma from head and neck [77] and in prostate cancer patients [78]. On the contrary, it was found down-regulated in colorectal [79] and esophageal cancer [80]. MiR-720 was described to inhibit breast tumor invasion and migration by targeting the metastasis promoter TWIST1 [81]. Conversely, it resulted hyper-expressed in colorectal cancer [82].

MiR-125a-5p is transcribed as a cluster with let-7 and miR-199b. Similarly to miR-99b, miR-125a-5p revealed hypo-expression during the treatment. On the other hand, it increased and showed over-expression in tumors. Interestingly, miR-125a-5p differential expression, detected on pooled RNAs, is maintained with statistical significance in mice HF fed for 3, 6, and 12 months individually analyzed, and in tumors, suggesting miR-125a-5p potential high impact at the functional level starting from the early stage of the liver disease. MiR-125a-5p seems to possess oncogenic or tumor suppressor activities. It was described as an epidermal growth factor signaling-regulated miRNA which can negatively regulate human lung cancer cell migration and invasion *in vitro*, is frequently down-regulated in lung cancer [83], and seems to play a role in enhancing *in vitro* cell migration and invasion in NSCLC [84]. Yang et al. [85] described miR-125a-5p up-regulated in lung squamous cell carcinoma (SCC), whereas miR-125a-5p low expression levels in tissues or serum have been associated with enhanced malignant potential in gastric and breast cancer [86, 87]. MiR-125a-5p was described over-expressed in thyroid carcinomas [88] and hypo-expressed in human HCC [89], where it was shown to target the 3′UTR of *SIRT7*, a member of the Sirtuin family, whose activity in cancer, ER and genomic stress response, hepatosteatosis has already been investigated and is still controversial [90]. Two predicted miR-125a-5p interaction sites are also detected on mouse *SIRT7* (microRNA.org: mirSVR score −0.0006, −0.3021; PhastCons 0.5087, 0.5259, respectively). MiR-125a-5p is also involved in lipid metabolism [91] and its level has been found increased in hyperlipidemic and/or hyperglycemic patients’ sera [92]. MiR-125a-5p serum levels have been also described as biomarkers in liver diseases [93].

MiR-182 showed over-expression already after 3 months of HF diet, and this trend was markedly maintained in mice, also at the individual level, during the treatment and in tumors. Several studies demonstrated miR-182 involvement in HCC and metastasis, by controlling the expression of genes with tumor suppressor activity, such as the metastasis suppressor *MTSS1* [94], *Cebpa* [95], *ephrinA5* [96], and *FOXO1* [97]. MiR-182 was also down-regulated in fibrosis related to NAFLD, where *FOXO3* was described as a target [98]. MiR-182/*Cebpa/ephrinA5/FOXO1/FOXO3* alignments can be also predicted on *Mus musculus*, putatively indicating a role of miR-182 in the regulation of those genes. Our data demonstrate early involvement of miR-182 in the transition of liver injury, which is maintained up to HCC initiation and development, indicating that early deregulation of this microRNA could be one among the factors putatively responsible for the hepatic disease here represented, and for its progression.

## Conclusions

In this study, based on the sequential analysis of the progression of HF diet-induced hepatic damage through NAFLD-NASH-HCC in a long term-fed mouse model, fifteen microRNAs were described to be modulated and differentially expressed in hepatic tissues and in tumors, providing a “signature” in the transition of liver injury until HCC development. A number of dysregulated microRNAs in this model were already described in the pathogenesis of liver disease and showed concordant level of expression with respect to that already described in literature (miR-155, miR-20a, miR-182, miR-200a, miR-200c, miR-27a, miR-31, miR-99b) or discordant expression level (miR-193b, miR-93, miR-125a-5p). Four dysregulated microRNAs (miR-340-5p, miR-484, miR-574-3p, miR-720), never described in liver damage and tumorigenesis, were here detected. Interestingly, two miRNAs (miR-125a-5p and miR-182) showed significant early dysregulation, indicating a putative role and involvement starting from the first stages of the liver disease. In conclusion, the study provides new information about dysregulated microRNAs in diet-induced liver damage and hepatocarcinogenesis. Additional functional analyses are needed to validate the results here obtained, and to better define the role of these molecules in the progression of the hepatic disease.

## Additional files

Additional file 1: Figure S1. Inflammatory infiltrate. Lymphocytes (green arrows), plasma cells (yellow arrows), macrophages (red arrows), and PMN (blue arrow). (PPTX 1448 kb) Additional file 2: Figure S2. MiRNAs differentially modulated during the progression of the hepatic damage. (A) RQ (relative quantification) values ± SE (Y axis) obtained by comparing HF to LF pooled RNAs from hepatic tissues. (B) RQ values ± SE (Y axis) of pooled RNAs from tumor tissues with respect to pooled RNAs from HF hepatic non-tumor tissues. Results are from 3 replicates. Global normalization mode was used for the analysis. Samples marked with the asterisk show *P* ≤ 0.05. (PDF 284 kb)

## Abbreviations

ER: endoplasmic reticulum
HCC: hepatocellular carcinoma
HF: high fat
LF: low fat
miRNA: microRNA
NAFLD: non-alcoholic fatty liver disease
NASH: non-alcoholic steato-hepatitis
PMN: polymorphonuclear leukocytes
