# Supplementary material for: MicroRNA expression analysis in high fat diet-induced NAFLD-NASH-HCC progression: study on C57BL/6J mice
Source: BMC Cancer. 2016 Jan 5;16:3. doi: 10.1186/s12885-015-2007-1 (PMC4700747; doi:10.1186/s12885-015-2007-1)
Supplement: Additional file 1: Figure S1. — Inflammatory infiltrate. Lymphocytes (green arrows), plasma cells (yellow arrows), macrophages (red arrows), and PMN (blue arrow). (PPTX 1448 kb) [file 12885_2015_2007_MOESM1_ESM.pptx]

## Slide 1
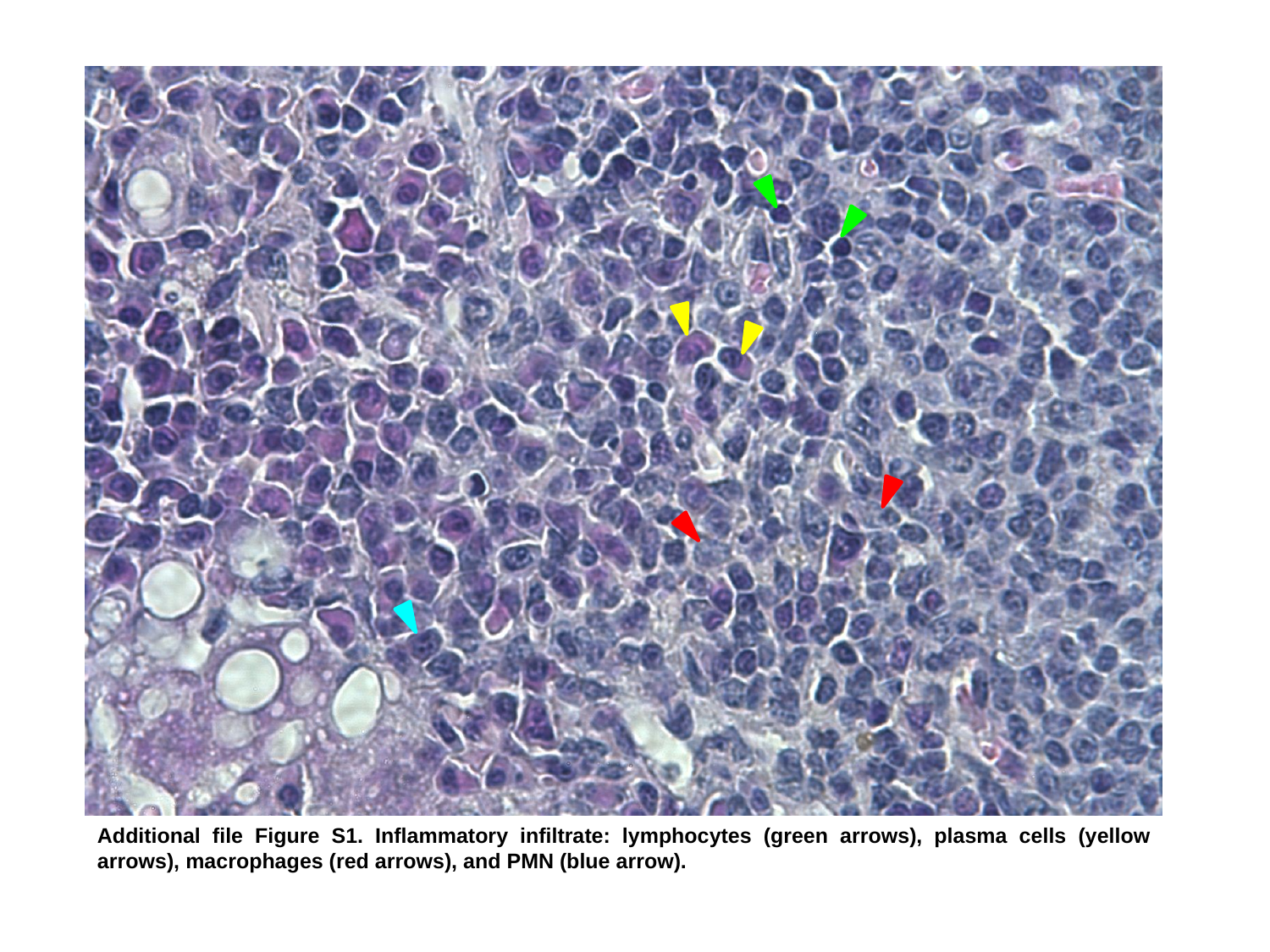

Additional file Figure S1. Inflammatory infiltrate: lymphocytes (green arrows), plasma cells (yellow arrows), macrophages (red arrows), and PMN (blue arrow).
