# Supplementary figures and images for: MicroRNA expression analysis in high fat diet-induced NAFLD-NASH-HCC progression: study on C57BL/6J mice
Source: BMC Cancer. 2016 Jan 5;16:3. doi: 10.1186/s12885-015-2007-1 (PMC4700747; doi:10.1186/s12885-015-2007-1)

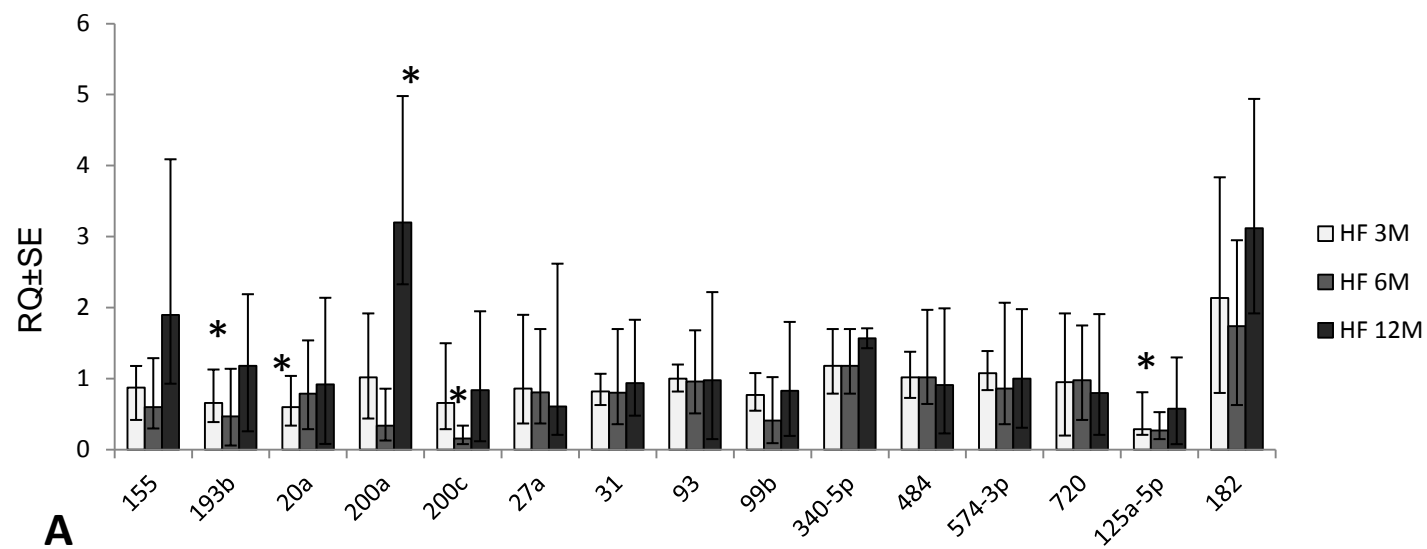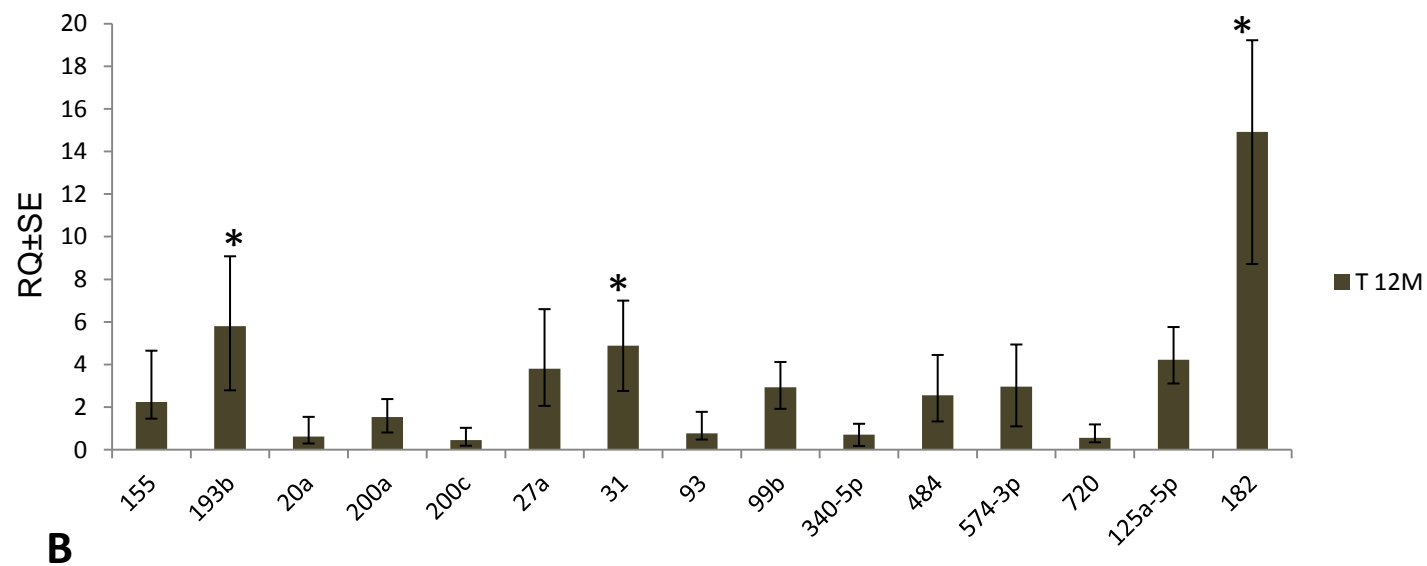

Supplement: Additional file 2: Figure S2. — MiRNAs differentially modulated during the progression of the hepatic damage. (A) RQ (relative quantification) values ± SE (Y axis) obtained by comparing HF to LF pooled RNAs from hepatic tissues. (B) RQ values ± SE (Y axis) of pooled RNAs from tumor tissues with respect to pooled RNAs from HF hepatic non-tumor tissues. Results are from 3 replicates. Global normalization mode was used for the analysis. Samples marked with the asterisk show P ≤ 0.05. (PDF 284 kb) [file 12885_2015_2007_MOESM2_ESM.pdf]
